# Prevalence and genetic diversity analysis of human coronaviruses among cross-border children

**DOI:** 10.1186/s12985-017-0896-0

**Published:** 2017-11-22

**Authors:** Peilin Liu, Lei Shi, Wei Zhang, Jianan He, Chunxiao Liu, Chunzhong Zhao, Siu Kai Kong, Jacky Fong Chuen Loo, Dayong Gu, Longfei Hu

**Affiliations:** 1 Department of Health Inspection and Quarantine, School of Public Health, Sun Yat-sen University, Guangzhou, 510275 People’s Republic of China; 2 Central Laboratory of Health quarantine, Shenzhen International Travel Health Care Center and Shenzhen Academy of Inspection and Quarantine, Shenzhen Entry-exit Inspection and Quarantine Bureau, Shenzhen, 518033 People’s Republic of China; 3 Wuhan Institute of Virology, Chinese Academy of Science, Wuhan, 430071 People’s Republic of China; 4 Biochemistry Programme, School of Life Sciences, The Chinese University of Hong Kong, Hong Kong, Hong Kong SAR China; 5 Department of Biomedical Engineering, The Chinese University of Hong Kong, Hong Kong, Hong Kong SAR China; 6 Shenzhen Academy of Inspection and Quarantine, Shenzhen, 518010 People’s Republic of China

**Keywords:** Human coronaviruses, Cross-border children, Molecular epidemiology, Phylogenetic analysis, Genetic diversity

## Abstract

**Background:**

More than a decade after the outbreak of human coronaviruses (HCoVs) SARS in Guangdong province and Hong Kong SAR of China in 2002, there is still no reoccurrence, but the evolution and recombination of the coronaviruses in this region are still unknown. Therefore, surveillance on the prevalence and the virus variation of HCoVs circulation in this region is conducted.

**Methods:**

A total of 3298 nasopharyngeal swabs samples were collected from cross-border children (<6 years, crossing border between Southern China and Hong Kong SAR) showing symptoms of respiratory tract infection, such as fever (body temperature > 37.5 °C), from 2014 May to 2015 Dec. Viral nucleic acids were analyzed and sequenced to study the prevalence and genetic diversity of the four human coronaviruses. The statistical significance of the data was evaluated with Fisher chi-square test.

**Results:**

78 (2.37%; 95%CI 1.8-2.8%) out of 3298 nasopharyngeal swabs specimens were found to be positive for OC43 (36;1.09%), HKU1 (34; 1.03%), NL63 (6; 0.18%) and 229E (2;0.01%). None of SARS or MERS was detected. The HCoVs predominant circulating season was in transition of winter to spring, especially January and February and NL63 detected only in summer and fall. Complex population with an abundant genetic diversity of coronaviruses was circulating and they shared homology with the published strains (99-100%). Besides, phylogenetic evolutionary analysis indicated that OC43 coronaviruses were clustered into three clades (B,D,E), HKU1 clustered into two clades(A,B) and NL63 clustered into two clades(A,B). Moreover, several novel mutations including nucleotides substitution and the insertion of spike of the glycoprotein on the viral surface were discovered.

**Conclusions:**

The detection rate and epidemic trend of coronaviruses were stable and no obvious fluctuations were found. The detected coronaviruses shared a conserved gene sequences in S and RdRp. However, mutants of the epidemic strains were detected, suggesting continuous monitoring of the human coronaviruses is in need among cross-border children, who are more likely to get infected and transmit the viruses across the border easily, in addition to the general public.

## Background

Human coronaviruses (HCoVs) have been causing worldwide outbreak with cases of hospitalization [1]. Six types of coronaviruses (CoVs) are known to infect human: two α-CoVs, i.e. 229E and NL63, two β-CoVs group A, i.e. HKU1 and OC43, β-CoVs group B, i.e. Severe Acute Respiratory Syndrome Coronavirus (SARS-CoV) and β-CoVs group C, i.e. Middle East Respiratory Syndrome Coronavirus (MERS-CoV). SARS-CoV and MERS-CoV, which are highly pathogenic to human lives and have caused serious diseases or death, causes about 10 and 36% mortality respectively. OC43, HKU1, NL63 and 229E are the most common four HCoVs in most regions, circulating worldwide with a detection rate ranging from 1.1 - 8.5% and with variations in their predominantly circulating seasons and strains [2–5]. HCoVs ranks the third in the detection rate of all 17 respiratory viruses in south of China (Guangzhou) and poses a heavy burden to the health care of children as it is associated with acute upper or lower respiratory tract infections, and cases of death have been reported [6]. Moreover, high mutation rates caused by the low fidelity of RNA-dependent RNA polymerase (RdRp) led to high diversity of HCoVs [7]. Several studies about the genetic diversity of human coronaviruses on hospitalized patients had been carried out previously. The new OC43 genotype D based on the recombination of B and C was discovered in 2005 [8]. Two additional recombinants: E (CH) and E (FR) were reported as homologous genome recombination in 2015 [9, 10]. The genetic features of NL63 were reported at least three distinct circulating genotypes (A, B and C) and one recombinant (cluster R) in the United States in 2011 [11]. Meanwhile, HKU1 strains were grouped into three clusters (A, B and C) due to natural recombination [12]. These previous reports focused on hospitalized patients, who have low mobility and seldom cross the border, while this study hereby firstly reports the analysis on cross-border children, mainly including “cross-boundary students”, who are born and attend school in Hong Kong but reside in Mainland China [13, 14]. A border still exists between Shenzhen in Mainland China and Hong Kong (SZ-HK port) due to the colonial history, resulting in different health care and education systems [13]. Children had a high incidence of coronaviruses infection and “cross-boundary students” connecting closely Hong Kong and Mainland China will help us understand the epidemic characteristics of coronaviruses in the Pearl River Delta region. New occurrence of infectious coronaviruses and the known pan-coronavirus variation among this region are of our study interest because the coronaviruses have the potential to threaten global health system and no vaccine is currently available [15, 16]. Therefore, surveillance upon human coronaviruses among this region was carried in this study.

## Methods

### Clinical specimens collection

This was a cross-sectional study in molecular epidemiology for coronaviruses infection, and the minimum sample size of this study was 1683 as determined by Z distribution. A total of 3298(>1683) nasopharyngeal swabs samples were collected from children (<6 years) who passed Shenzhen border, linking Southern China and Hong Kong SAR, from 2014 to 2015 and showed symptoms of respiratory tract infection, such as fever (body temperature > 37.5 °C) and cough. Written informed consent was obtained from the guardians of all participants before the sample and data collection.

### Sample preparation

Briefly, nasopharyngeal swab was collected and stored in a sterile EP tube with 5 mL viral transport medium in Shenzhen border. All the samples collected were immediately refrigerated at 2-8 °C and transported to the central laboratory of health quarantine of Shenzhen Entry-exit Inspection and Quarantine Bureau (SZCIQ) within the same day and stored at −80 °C until analysis.

### Molecular screening of virus and amplification, sequencing of RdRp and S genes

Viral nucleic acids were extracted from 200 μL respiratory samples using MagNA pure 96 DNA with Viral NA small volume kit (Roche) and EZ1 virus Mini kit V2.0 (Qiagen) according to the manufacturer’s instructions. The viral nucleic acids were stored at −80 °C until use. For the coronaviruses screening, a quantitative real-time polymerase chain reaction (qRT-PCR) was performed in triplicate using ABI 7500 qRT-PCR thermocycler. The specimens were firstly screened for influenza viruses according to the procedure previously published [17]. Samples of negative results on influenza were then tested for pan-coronavirus as well as 13 other common respiratory viruses. The qRT-PCR master mixture was performed according to the manufacturer’s instructions of qRT-PCR Kit (Quant), mainly contained 20.0 μL buffer and 5.0 μL RNA. The thermal cycling conditions were set as follows: reverse transcription at 50 °C for 10 min, initial 95 °C for 3 min, 40 cycles of PCR amplification at 95 °C for 15 s, annealing/elongation at 60 °C for 45 s. The partial S (S1 subunit) and RdRp genes were detected in the positive samples after HCoVs screening with the forward (F) and reverse (R) primers listed in Table 1. The PCR mixture (25 μL) contained 5.0 μL of RNA, PCR buffer mixed with Superscript ®III/PT Taq Kit (Invitrogen) containing 12.5 μl of 2× Rxn Mix,1 μL of forward and reverse primer (10 μM), 1.0 μL of MgSO_4_, 1.0 μL of BSA (0.1%),1.0 μl of Superscript ®III/PT Taq Enzyme, 0.5 μL of RNA Inhibitor, 2.0 μL of nuclease free water. The thermal cycling conditions were set as follows: reverse transcription at 50 °C for 30 min, 35 cycles of PCR amplification at 94 °C for 30 s, annealing at 50–54 °C for 30 s, elongation at 68 °C for 150–180 s, final elongation at 68 °C for 5 min. Sanger sequencing (Sangon Biotech) of the PCR products of concentration ranging from 50 to 300 ng/μL was performed to study the homology and mutations of samples. Genetic sequence data have been submitted to a publicly available repository (Genbank) and the accessible sequence accession numbers (MF996589-MF996664) including features of the samples and sequences.

**Table 1:** PCR primers of RdRp, S genes of four HCoVs

Target genes	HCoVs	Primer	Sequence (5′- 3′)	Location
RdRp	OC43	F	CGAGTGTAGATGCCCGTCTCG	13,353-13,373
		R	GCATCTGTCTTAACAACATCATC	15,990-15,970
	HKU1	F	GAATGCCCGGCTAGTACCCTGTGC	13,581-13,604
		R	GGGTAAGCATCTATAGCTAGAC	16,127-16,106
	NL63	F	GGCACGGACATCGATAAGTGTG	112,481-12,505
		R	GCATCTGTCTTAACAACATCATC	14,954-14,932
	229E	F	CTGAAGTCCAATTGTGTGCGC	12,493-12,513
		R	CACCTTCGTTAAGAGTCTTGTTGAG	15,034-15,010
S	OC43	F	TCCCTGATTTACCCATTTGTG	23,486-23,506
		R	ATAGTTAATGGGTTGCAGCTGT	25,807-25,786
	HKU1	F-1	TATGTTAATAAWACTTTGTATAGTG	23,236-23,260
		R-1	TACAATTGACAAGAACTAGAAG	24,179-24,158
		F-2	ACCTCTTAATTGGGAACGTA	23,922-23,941
		R-2	GAAGATCTCTAATTTCACTACCAC	25,717-25,694
	NL63	F	GAGTGGTAGGTTGTTGTTACGCAATAATGG	20,403-20,432
		R	GTCACGCAAGACAGTAACATCATGAGGTGG	24,643-24,614

### Statistical and sequence analysis

The statistical significance of the data was evaluated with SPSS 20.0. All the *p*-value determined by Fisher’s Chi-square test and a *p*-value <0.05 was considered statistically significant. DNASTAR was used to analyze and illustrate the gene sequences compared with the sequences in NCBI Genbank for homology study. The phylogenetic trees were constructed by MEGA 7.0 with the best bases substitution model consideration, neighbour-joining, maximum likelihood and bootstrap values adjustment.

## Results

**Fig. 1:**
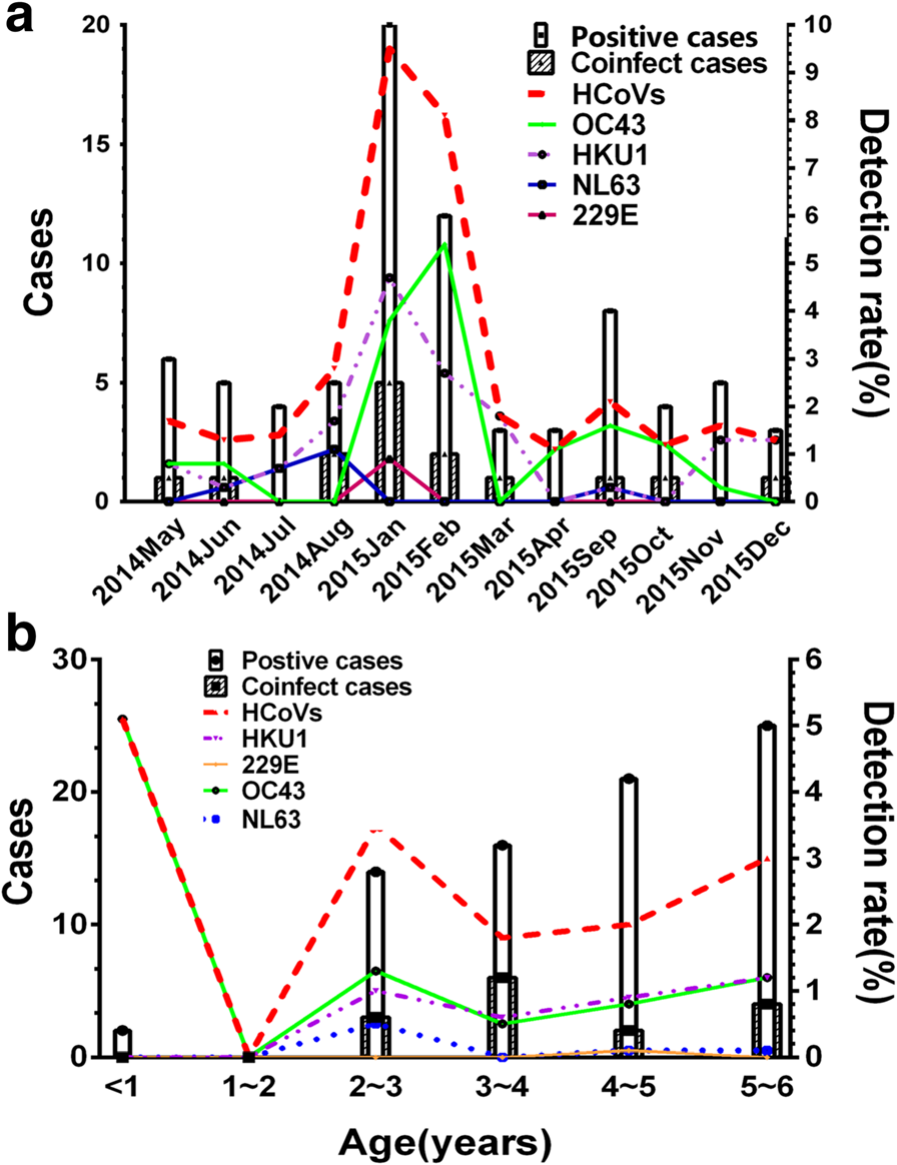
Epidemiological characteristics of human coronaviruses infection among Cross-border children. **a** Distribution of the four HCoV infections based on Month group. **b** Distribution of the four HCoV infections based on Age group. Positive and co-infected cases were plotted on the left Y-axis and others were plotted on the right Y-axis. Different strains or total HCoVs were indicated according to the key

Three thousand, two hundred and ninety-eight nasopharyngeal swabs samples were screened to study the prevalence and clinical characteristics of HCoVs infection. All the coronaviruses detected in this study could be typed. 78 (2.37%; 95%CI 1.8- 2.8%) out of 3298 nasopharyngeal swabs specimens were found to be positive for OC43 (36; 1.09%; 95% CI 0.74%-1.44%), HKU1 (34; 1.03%; 95%CI 0.69%-1.37%), NL63 (6; 0.18%; 95%CI 0.04%-0.32%) and 229E (2; 0.01%) and none of SARS and MERS were detected. The HCoVs predominant circulating season was in transition of winter to spring, especially January and February and NL63 detected only in summer and fall (Fig. 1a). The results of the clinical symptoms of these samples were shown in Table 2. Males and females shared a common detection rate of all the HCoVs studied and no significant difference was found among the detection rate of the four strains. Also, the *p* values of Fisher’s chi-square test showed no significant difference in detection rates among different origins. The first three clinical symptoms of HCoVs infection were fever (*p* = 0.08), throat congestion (*p* = 0.58) and antiadoncus (*p* = 0.09). Yet, there was no significant difference between HCoVs infected and non-infected patients. For the age group distribution of four HCoVs infections, the infant age group (<1 year old) with weaker respiratory immunity was showed with the highest infection rate in total types of HCoVs infection *(p = 0.049)* and OC43 infection *(p = 0.068)*(Fig. 1b). There was virus co-infection between human coronaviruses with other common respiratory diseases. Adenovirus(Adv) and Rhinovirus(RV) were the most common two viruses that concomitantly detected with HCoVs in children younger than 6 years old.

**Table 2:** Statistics of HCoVs Infected and Non-Infected Children

	Parameter	No. (%) of non-HCoVs	No. (%) of HCoVs				
			OC43 (*n* = 36)	HKU1 (*n* = 34)	NL63 (*n* = 6)	229E (*n* = 2)	Total (*n* = 78)
Gender	Male	2184(97.6)	22(1.0)	25(1.1)	5(0.2)	1(0.0)	53(2.4)
	Female	1036(97.6)	14(1.4)	9(0.9)	1(0.1)	1(0.1)	25(2.4)
Symptom	Hypothermia	70(2.2)	0(0.0)	0(0.0)	0(0.0)	0(0.0)	0(0.0)
	Fever	3060(95.0)	36(100.0)	34(100.0)	6(100.0)	2(100.0)	78(100.0)
	Ardent fever	305(9.5)	2(5.6)	2(5.9)	0(0.0)	0(0.0)	4(5.1)
	Running nose	412(12.8)	5(13.9)	5(14.7)	1(16.7)	0(0.0)	11(14.1)
	Cough	883(27.4)	9(25.0)	4(11.8)	0(0.0)	1(50.0)	14(17.9)
	Throat congestion	1466(45.5)	15(41.7)	18(52.9)	3(50.0)	2(100.0)	38(48.7)
	Nasal obstruction	36(1.1)	0(0.0)	0(0.0)	0(0.0)	0(0.0)	0(0.0)
	Antiadoncus	671(20.8)	7(19.4)	12(35.3)	2(33.3)	0(0.0)	21(26.9)
	Diarrhea	16(0.5)	0(0.0)	0(0.0)	0(0.0)	0(0.0)	0(0.0)
	Flush	28(0.9)	1(2.8)	0(0.0)	0(0.0)	0(0.0)	1(1.3)
	Vomiting	34(1.0)	0(0.0)	0(0.0)	0(0.0)	0(0.0)	0(0.0)
	Hemoptysis	1(0.0)	0(0.0)	0(0.0)	0(0.0)	0(0.0)	0(0.0)
	Rash	7(0.2)	0(0.0)	0(0.0)	0(0.0)	0(0.0)	0(0.0)
	None	38(1.2)	0(0.0)	0(0.0)	0(0.0)	0(0.0)	0(0.0)
Region	Mainland China	2741(97.5)	31(1.1)	32(1.1)	4(0.1)	2(0.1)	69(2.5)
	Hong Kong	438(98.9)	2(0.5)	2(0.5)	1(0.2)	0(0.2)	5(1.1)
	Others	41(91.1)	3(6.7)	0(0.0)	1(2.2)	0(0.0)	4(8.9)

**Fig. 2:**
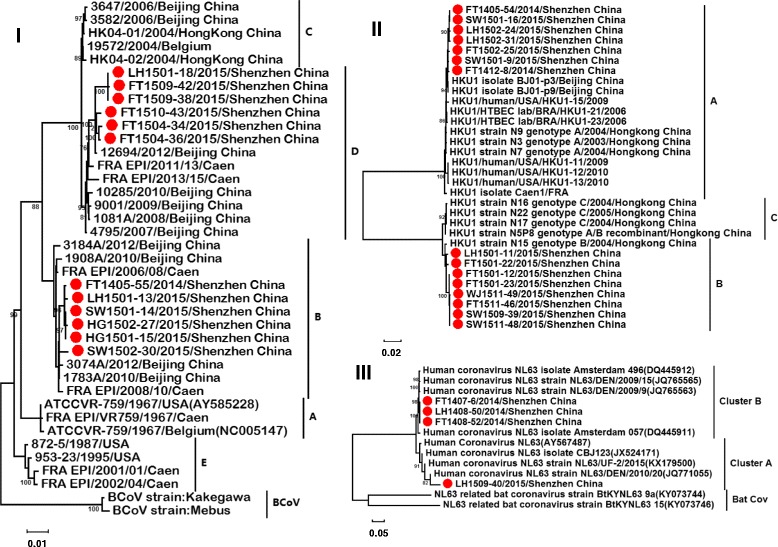
Phylogenetic analysis based on partial S genes of OC43, HKU1 and NL63. (I) Phylogenetic tree of OC43 S genes (2.2 kb) constructed with maximum likelihood; (II) Phylogenetic tree of HKU1 S genes (2.4 kb) constructed with maximum likelihood; (III) Phylogenetic tree of NL63 S genes (4.0 kb) constructed with neighbour-joining. Our samples were indicated with a red spot and others were used as referenced strains from complete genomes in GenBank. The strains indicated with “*” were clustered into genotype E, recombinant of B, C and D. The OC43 and NL63 phylogenetic trees were constructed using BCoV and Bat CoV respectively as outgroup

A total of 40 RdRp genes, including 20 for OC43, 15 for HKU1, 4 for NL63 and 1 for 229E, and 36 S genes, including 16 for OC43, 16 for HKU1 and 4 for NL63, were sequenced to perform phylogenetic analysis. Since there is a high conservative in RdRp gene, phylogenetic tree was not shown here. Multiple alignments results of RdRp genes indicated that OC43 and HKU1 possessed 99–100% nt identities. Largest divergences were observed in HKU1 coronaviruses, which possessed 96 - 100% nt identities, but sequences detected in this study were 99-100% homologous to the published strains (Table 3). For the phylogenetic trees constructed based on 31 S genes with a genomic length over 2 kb of four HCoVs, there was a high level of genetic diversity among those HCoVs (Fig. 2). The OC43 coronaviruses were clustered into clade B (5,41.7%), clade D (6,50%) and clade E(1,8.3%) while none of the strains of genotype A and C was detected (Fig. 2I). Besides, there was one OC43 sequence (SW1502-30/2015/Shenzhen, China) being clustered with a new recombination genotype E (CH) (Genbank accession no: KP198611.1). Similarly, HKU1 strains in this study were clustered into clade A (7,46.7%) and clade B (8,53.3%) and related to the sequences detected in Beijing and Hong Kong SAR respectively, while no clade C was detected (Fig. 2 II). NL63 strains in this study were clustered into clade A (1,25.0%) and clade B (3,75.0%), related to strains isolated from USA and Denmark, while no clade C were detected neither (Fig. 2 III).

**Table 3:** Statistics of closely related strains of HCoVs based on RdRp and S gene

Based	HCoVs	Closely related strains	Homology (%)	No. (%)
RdRp gene	OC43	Human coronavirus OC43 isolate 12,694/2012 (genotype D, Beijing)	99-100	16 (80.0)
		Human coronavirus OC43 isolate 5617/2007 (genotype D, Beijing)	99-100	2 (10.0)
		Human coronavirus OC43 isolate 5595/2007 (genotype D, Beijing)	99	2 (10.0)
	HKU1	Human coronavirus HKU1 isolate BJ01-p9 (genotype A, Beijing)	99	7 (46.7)
		Human coronavirus HKU1 strain N15 (genotype B, Hong Kong)	99	8 (53.3)
	NL63	Human coronavirus NL63 strain NL63/human/USA/0111-25/2001 (USA)	99	3 (75.0)
		Human coronavirus NL63 isolate NL63/UF-2/2015 (USA)	99	1 (25.0)
	229E	Human coronavirus 229E isolate HCoV-229E/BN1/GER/2015 (Germany)	99	1 (100.0)
S gene	OC43	Human coronavirus OC43 isolate 12,694/2012 (genotype D, Beijing)	99	10 (62.5)
		Human coronavirus OC43 isolate 3184A/2012 (genotype B, Beijing)	99	6 (37.5)
	HKU1	Human coronavirus HKU1 isolate BJ01-p9 (genotype A, Beijing)	99	8 (50.0)
		Human coronavirus HKU1 strain N15 (genotype B, Hong Kong)	99	8 (50.0)
	NL63	Human coronavirus NL63 strain NL63/human/0111-25/2001/USA	99	3 (75.0)
		Human coronavirus NL63 strain NL63/DEN/2009/20/Denmark	99	1 (25.0)

**Fig. 3:**
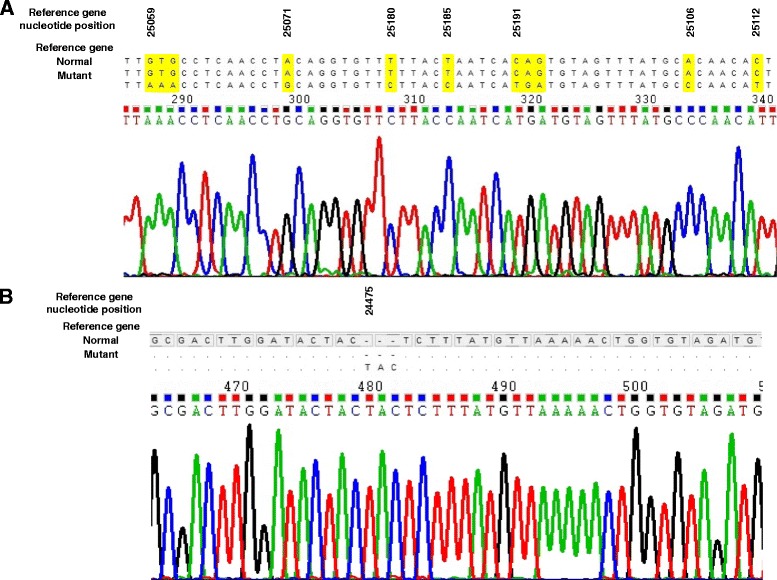
Mutation analysis on the S genes of OC43 and HKU1. **a** Bases substitution in S1 genes of OC43. **b** Extra insertion in putative RBD of HKU1

Moreover, we found nucleotide mutations in some of the samples (Fig. 3). Three out of 8 OC43 coronaviruses of genotype D had a total of 11 bases substitution in nucleotide position 25,059–25,112 of S genes (Genbank accession number of referenced strain: KF923904.1) (Fig. 3a). Six out of 8 HKU1 coronaviruses of genotype B were found with an extra insertion in nucleotide position 24,465 of genome leading to an additional amino acid “Threonine” insertion in amino acid position 510 of Spike (Genbank accession of referenced strain: DQ415911.1) (Fig. 3b).

## Discussion

The detection rate of total HCoVs was 2.37% (95% CI: 1.8 to 2.8%) in this study was consistent with the previous studies. All the coronaviruses detected have been typed. OC43 was the most common coronaviruses in our study consistent with reports in Guangzhou, Hong Kong, USA and England [4, 18–20], but some studies demonstrated that the prevalence of NL63 was similar to or even higher than that of OC43 in Brazil, Kenya and Japan [3, 21–23]. 229E was detected in low levels throughout years as previous reports and thus the peak activity of 229E could not be determined. The HCoVs predominant circulating season was in transition of winter to spring, especially January and February. NL63 predominant circulating seasons were summer and fall, which were different from those reports of winter and spring in temperate countries, such as the USA and Netherlands [24, 25]. None of the infection was found in the 1–2 years old group, even though the number of sample of this group was higher than that of the infant age group. In summary, we had analyzed the prevalent and clinical characteristics of HCoVs infection in cross-border children in SZ-HK ports. Compared with previous reports, the detection rate and epidemic trend of coronaviruses were stable, and no obvious fluctuations were found. Yet, none of novel infectious coronaviruses, SARS and MERS were detected in this study.

The coronaviruses detected from SZ-HK ports had a high homology with the published strains indicated a stable gene sequences in S and RdRp. However, there were great genetic diversity among these circulating strains. OC43 detected in this report cluster with genotype B, D and E strains, while none of genotypes A and C were detected, probably because genotype A strains had disappeared and genotype C strains were not included in this study [9]. We observed six OC43 coronaviruses were closely related to the genotype B detected from Beijing based on S genes. It possessed 99% nt identities and showed an incongruent phylogenetic relationship between RdRp and S genes. New Recombination genotypes led by high intra-specific diversity have been reported in studying OC43 coronaviruses circulating in France, where eight different recombinants were discovered and confirmed with in silico analysis of complete genomes available using partial genome sequencing [10]. At present, the base substitution and insertion in OC43 and HKU1 is novel and could not find any matches in either OC43 or HKU1 strains in Genbank library. More importantly, these amino acid sites are located in one of the putative regions of HKU1 receptor binding domain [26]. The protein structure and its related function, especially on the efficiency on human infection, need to be investigated in the future.

## Conclusions

The detection rate of coronaviruses were in line with previous reports, no novel infectious coronaviruses was detected, the epidemic trend of coronaviruses were stable and all the infectors showed normal respiratory infection symptoms. Besides there were great genetic diversity of coronaviruses detected from SZ-HK ports and all the strains had a high homology compared with the published strains. However, mutant of the epidemic strains detected during our surveillance are increasing, therefore continuous monitoring of the human coronaviruses is in need among cross-border children, who are more likely to get infected and transmit the viruses across the border easily, in addition to the general public.

## Abbreviations

Adv: Adenovirus
Bat CoV: Bat coronavirus
BCoV: Bovine coronavirus
Cox A6: Coxsackievirus A6
EV: Enterovirus
HBoV: Human Bocavirus
HCoVs: Human coronaviruses
MP: Mycoplasma pneumonia
qRT-PCR: Quantitative real-time polymerase chain reaction
RdRp: RNA-dependent RNA polymerase
RSV: Respiratory syncytial virus
RT-PCR: Reverse transcription polymerase chain reaction
RV: Rhinovirus
SZ-HK: Shenzhen-Hong Kong

## References

[1] Centers for Disease Control and Prevention. About Coronavirus. https://www.cdc.gov/coronavirus/about/index.html. Accessed 16 Apr 2017.

[2] World Health Organization. Middle East respiratory syndrome coronavirus (MERS-CoV). http://www.who.int/emergencies/mers-cov/en/. Accessed 16 Apr 2017.

[3] Cabeca TK, Granato C, Bellei N (2013). Epidemiological and clinical features of human coronavirus infections among different subsets of patients. Influenza Other Respir Viruses.

[4] Dijkman R, Jebbink MF, Gaunt E, Rossen JW, Templeton KE, Kuijpers TW, van der Hoek L (2012). The dominance of human coronavirus OC43 and NL63 infections in infants. J Clin Virol.

[5] Amini R, Jahanshiri F, Amini Y, Sekawi Z, Jalilian FA (2012). Detection of human coronavirus strain HKU1 in a 2 years old girl with asthma exacerbation caused by acute pharyngitis. Virol J.

[6] Liu WK, Liu Q, Chen DH, Liang HX, Chen XK, Chen MX, Qiu SY, Yang ZY, Zhou R (2014). Epidemiology of acute respiratory infections in children in Guangzhou: a three-year study. PLoS One.

[7] Woo PC, Lau SK, Huang Y, Yuen KY (2009). Coronavirus diversity, phylogeny and interspecies jumping. Exp Biol Med (Maywood).

[8] Vijgen L, Keyaerts E, Lemey P (2005). Circulation of genetically distinct contemporary human coronavirus OC43 strains. Virol J.

[9] Zhang Y, Li J, Xiao Y, Zhang J, Wang Y, Chen L, Paranhos-Baccala G, Ren L, Wang J (2015). Genotype shift in human coronavirus OC43 and emergence of a novel genotype by natural recombination. J Inf Secur.

[10] Kin N, Miszczak F, Lin W, Gouilh MA, Vabret A, EPICOREM Consortium (2015). Genomic analysis of 15 human Coronaviruses OC43 (HCoV-OC43s) circulating in France from 2001 to 2013 reveals a high intra-specific diversity with new recombinant genotypes. Viruses.

[11] Dominguez SR, Sims GE, Wentworth DE, Halpin RA, Robinson CC, Town CD, Holmes KV (2012). Genomic analysis of 16 Colorado human NL63 coronaviruses identifies a new genotype, high sequence diversity in the N-terminal domain of the spike gene and evidence of recombination. J Gen Virol.

[12] Woo PC, Lau SK, Yip CC, Huang Y, Tsoi HW, Chan KH, Yuen KY (2006). Comparative analysis of 22 coronavirus HKU1 genomes reveals a novel genotype and evidence of natural recombination in coronavirus HKU1. J Virol.

[13] Cross-Boundary Students - Hong Kong Special Administrative Region Government Press Releases. http://www.info.gov.hk/gia/general/201106/15/P201106150120.htm. Accessed 16 Apr 2017.

[14] Overview of the Health Care System in Hong Kong - Hong Kong Special Administrative Region Government portal: http://www.gov.hk/en/residents/health/hosp/overview.htm. Accessed 16 Apr 2017.

[15] Al-Tawfiq JA, Zumla A, Gautret P, Gray GC, Hui DS, Al-Rabeeah AA, Memish ZA (2014). Surveillance for emerging respiratory viruses. Lancet Infect Dis.

[16] Geller C, Varbanov M, Duval RE (2012). Human Coronaviruses: insights into environmental resistance and its influence on the development of new antiseptic strategies. Viruses.

[17] Loo JF, Wang SS, Peng F, He JA, He L, Guo YC, Gu DY, Kwok HC, Wu SY, Ho HP, Xie WD, Shao YH, Kong SK (2015). A non-PCR SPR platform using RNase H to detect MicroRNA 29a-3p from throat swabs of human subjects with influenza a virus H1N1 infection. Analyst.

[18] Gaunt ER, Hardie A, Claas EC, Simmonds P, Templeton KE (2010). Epidemiology and clinical presentations of the four human coronaviruses 229E, HKU1, NL63, and OC43 detected over 3 years using a novel multiplex real time PCR method. J Clin Microbiol.

[19] Woo PC, Yuen KY, Lau SK (2012). Epidemiology of coronavirus-associated respiratory tract infections and the role of rapid diagnostic tests: a prospective study. Hong Kong Med J.

[20] Prill MM, Iwane MK, Edwards KM (2012). Human coronavirus in young children hospitalized foracute respiratory illness and asymptomatic controls. Pediatr Infect Dis J.

[21] Cabeca TK, Carraro E, Watanabe A, Granato C, Bellei N (2012). Infections with human coronaviruses NL63 and OC43 among hospitalised and outpatient individuals in Sao Paulo, Brazil. J Mem Inst Oswaldo Cruz.

[22] Matoba Y, Abiko C, Ikeda T, Aoki Y, Suzuki Y, Yahagi K, Matsuzaki Y, Itagaki T, Katsushima F, Katasushima Y, Mizuta K (2015). Detection of the human coronavirus 229E, HKU1, NL63, and OC43 between 2010 and 2013 in Yamagata, Japan. Jpn J Infect Dis.

[23] Sipulwa LA, Ongus JR, Coldren RL, Bulimo WD (2016). Molecular characterization of human coronaviruses and their circulation dynamics in Kenya, 2009–2012. Virol J.

[24] Heald-Sargent T, Gallagher T (2012). Ready, set, fuse! The coronavirus spike protein and acquisition of fusion competence. Viruses.

[25] Pfefferle S, Oppong S, Drexler JF, Gloza-Rausch F, Ipsen A, Seebens A, Muller MA, Anna A, Vallo P, Adu-Sarkodie Y, Kruppa TF, Drosten C (2009). Distant relatives of severe acute respiratory syndrome coronavirus and close relatives of human coronavirus 229E in bats ,Ghana. Emerg Infect Dis.

[26] Qian Z, Ou X, Góes LGB, Osborne C, Castano A, Holmes KV, Dominguez SR (2015). Identification of the receptor-binding domain of the spike glycoprotein of human Betacoronavirus HKU1. J Virol.

